# The Degree of Early Life Stress Predicts Decreased Medial Prefrontal Activations and the Shift from Internally to Externally Guided Decision Making: An Exploratory NIRS Study during Resting State and Self-Oriented Task

**DOI:** 10.3389/fnhum.2013.00339

**Published:** 2013-07-03

**Authors:** Takashi Nakao, Tomoya Matsumoto, Machiko Morita, Daisuke Shimizu, Shinpei Yoshimura, Georg Northoff, Shigeru Morinobu, Yasumasa Okamoto, Shigeto Yamawaki

**Affiliations:** ^1^Department of Psychology, Graduate School of Education, Hiroshima University, Hiroshima, Japan; ^2^Institute of Biomedical and Health Sciences, Hiroshima University, Hiroshima, Japan; ^3^Faculty of Medicine, Hiroshima University, Hiroshima, Japan; ^4^Faculty of Psychology, Otemon Gakuin University, Osaka, Japan; ^5^Institute of Mental Health Research, University of Ottawa, Ottawa, ON, Canada

**Keywords:** internally guided decision making, very low-frequency fluctuations, task positive network, eyes-closed resting state, lateral prefrontal cortex, cortisol, mediation analysis, moderation analysis

## Abstract

Early life stress (ELS), an important risk factor for psychopathology in mental disorders, is associated neuronally with decreased functional connectivity within the default mode network (DMN) in the resting state. Moreover, it is linked with greater deactivation in DMN during a working memory task. Although DMN shows large amplitudes of very low-frequency oscillations (VLFO) and strong involvement during self-oriented tasks, these features’ relation to ELS remains unclear. Therefore, our preliminary study investigated the relationship between ELS and the degree of frontal activations during a resting state and self-oriented task using near-infrared spectroscopy (NIRS). From 22 healthy participants, regional hemodynamic changes in 43 front-temporal channels were recorded during 5 min resting states, and execution of a self-oriented task (color-preference judgment) and a control task (color-similarity judgment). Using a child abuse and trauma scale, ELS was quantified. We observed that ELS showed a negative correlation with medial prefrontal cortex (MPFC) activation during both resting state and color-preference judgment. In contrast, no significant correlation was found between ELS and MPFC activation during color-similarity judgment. Additionally, we observed that ELS and the MPFC activation during color-preference judgment were associated behaviorally with the rate of similar color choice in preference judgment, which suggests that, for participants with higher ELS, decisions in the color-preference judgment were based on an external criterion (color similarity) rather than an internal criterion (subjective preference). Taken together, our neuronal and behavioral findings show that high ELS is related to lower MPFC activation during both rest and self-oriented tasks. This is behaviorally manifest in an abnormal shift from internally to externally guided decision making, even under circumstances where internal guidance is required.

## Introduction

By definition, early life stress (ELS) derives from adverse experiences during childhood and adolescence including physical, sexual, and maltreatment abuse (Brown et al., 2009). Demonstrably, ELS is associated with deficits in cognitive and affective function (Pechtel and Pizzagalli, 2011) and is a significant risk factor for mood and anxiety disorders later in life (Heim and Nemeroff, 2001; Heim et al., 2010; Schmidt et al., 2011). Several lines of evidence have indicated that ELS elicits structural changes in the brain. For example, reports of some animal studies have described that ELS results in abnormally increased synaptic density in the infralimbic cortex (Ovtscharoff and Braun, 2001), and decreased dendritic spine density in the prefrontal cortex (PFC) (Murmu et al., 2006). Reports of human neuroimaging studies have described that ELS is associated with reduced gray matter volume including that of the PFC (De Bellis et al., 2002; Andersen et al., 2008; Paus et al., 2008; Hanson et al., 2010).

Although few functional neuroimaging studies have addressed the influence of ELS, activations within the default mode network (DMN) are known to be associated with ELS (Burghy et al., 2012; Philip et al., 2013a,b; van der Werff et al., in press; Cisler et al., 2013; Wang et al., in press). The DMN consists mainly of cortical midline structures (Northoff and Bermpohl, 2004; Raichle and Gusnard, 2005) and comprises the medial prefrontal cortex (MPFC), posterior cingulate cortex, and superior temporal/inferior parietal cortex (Fox et al., 2005; Kim et al., 2010; Qin and Northoff, 2011). The DMN is more active at rest than during goal-directed/externally guided cognitive tasks (Raichle et al., 2001; Buckner et al., 2008). Regions within the DMN show a high degree of functional connectivity during rest (Raichle et al., 2001; Beckmann et al., 2005; Raichle and Snyder, 2007; Buckner et al., 2008). Regarding these features of the DMN, ELS is known to be associated with greater deactivation of DMN during a working memory task (Philip et al., 2013b), and shows decreased functional connectivity within the DMN during a resting state (Burghy et al., 2012; van der Werff et al., in press; Cisler et al., 2013; Wang et al., in press; Philip et al., 2013a).

Neuronally, the DMN can be characterized by large amplitudes of spontaneous slow oscillations during a resting state (Raichle et al., 2001; Fransson, 2005; Zou et al., 2008). Slow oscillations have been observed using measurements of different types, functional magnetic resonance imaging (fMRI; Biswal et al., 1995; Fransson, 2006; Chepenik et al., 2010), electroencephalography (EEG; Horovitz et al., 2008; Helps et al., 2010; Broyd et al., 2011), and near-infrared spectroscopy (NIRS; Obrig et al., 2000; Näsi et al., 2011; Pierro et al., 2012). Slow oscillations from 0.04 to 0.15 Hz are called low-frequency oscillations (LFOs). Even lower frequency oscillations (<0.04 Hz) are designated as very low-frequency oscillations (VLFOs) (Obrig et al., 2000; Näsi et al., 2011). Although the mechanisms underlying the slow oscillations remain unclear, several reports of the literature have described these as neuronal characteristics of psychological personality traits (Kunisato et al., 2011) and psychiatric disorders such as anxiety (Hou et al., 2012) and mood disorders (Chepenik et al., 2010; Wang et al., 2012). Psychiatric disorders have shown high degrees of ELS (Heim and Nemeroff, 2001; Heim et al., 2010; Schmidt et al., 2011). Therefore, one would suspect high ELS to be related to changes in slow oscillations during the resting state. This point, however, remains to be investigated.

In addition to LFOs during the resting state, the DMN shows activation in fMRI during various tasks such as self-reference (Kelley et al., 2002; Northoff et al., 2006), episodic memory retrieval (Buckner et al., 2008), envisioning the future (Szpunar et al., 2007), mentalizing (Gusnard et al., 2001; Amodio and Frith, 2006), and internally guided decision making (Nakao et al., 2012). The DMN is often explained integratively as associated with self-oriented/internally guided psychological processes (Qin and Northoff, 2011; Whitfield-Gabrieli and Ford, 2012). Again, however, no report in the relevant literature has described the association between ELS and DMN activity during self-oriented tasks.

This preliminary study was undertaken to investigate the relations between ELS and the degree of MPFC activations during a resting state and self-oriented task using NIRS. This non-invasive technique uses near-infrared light to evaluate spatiotemporal characteristics of brain function near the brain surface. The use of NIRS enables the detection of spontaneous slow oscillations in oxygenated hemoglobin (oxy-Hb: Obrig et al., 2000). The LFOs and VLFO measured by NIRS are known to be differentiated from other oscillatory phenomena such as heart beat and respiratory cycles (Obrig et al., 2000). The activation of surface regions of MPFC during self-oriented tasks has also been measured using NIRS (Di Domenico et al., 2012).

**Figure 1:**
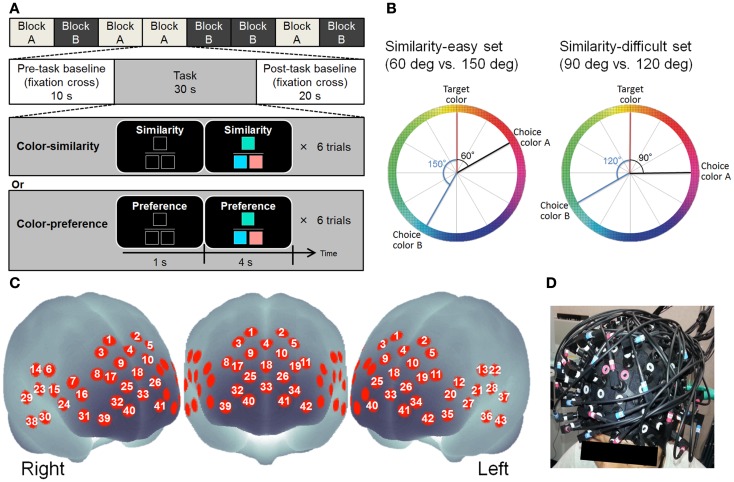
**(A)** Design of cognitive tasks. **(B)** Schematic figure showing how to make color combinations in the color-similarity judgment and color-preference judgment tasks. The left color wheel portrays examples of the color combinations of the similarity-easy set. The right color wheel displays examples of color combinations of the similarity-difficult set. The degrees from target color to choice color signify the color similarity. **(C)** Approximate location of the NIRS channel positions in MNI space. **(D)** NIRS probe position.

For the experiment described hereinafter, a child abuse and trauma scale (CATS) (Sanders and Becker-Lausen, 1995) was used to assess ELS. To control the effect of the recent stress level, we used the life event stress scale (LES) (Sarason et al., 1978). Stressful life events are known to affect brain function adversely through elevated cortisol level in the blood which is acutely or chronically caused by the hormonal stress response system: the hypothalamic–pituitary–adrenal (HPA) axis (Numakawa et al., 2013). Therefore, we also measured the blood levels of cortisol to assess whether early and/or recent life stress might elevate cortisol concentrations in the blood, resulting in alteration of PFC activation. We recorded eyes-closed (EC) and eyes-open (EO) resting-state NIRS before conducting cognitive tasks. In self-oriented cognitive and control tasks, color stimulus was used (see Figure 1A for example). The same color stimulus and color stimulus pairs were used in both tasks. As a self-oriented task, color-preference judgment (Johnson et al., 2005; Nakao et al., 2013) was used while the color-similarity judgment served as control (Johnson et al., 2005; Nakao et al., 2013) (see Figure 1A). We used these tasks for the following three reasons. First, using these tasks, we can differentiate between goal-directed/externally guided and self-oriented/internally guided psychological processes (Johnson et al., 2005; Nakao et al., 2013). Although color-similarity judgment requires participants to make a decision based on the external criterion (i.e., color-similarity), color-preference judgments require participants to make a decision based on their own internal criteria. Second, the same color-set is used in both tasks: the effects of stimuli can be well controlled. Third, Johnson et al. (2005) reported that the color-preference judgment activate the DMN including the MPFC [Brodmann area (BA) 9, 10] compared to the color-similarity judgment. The MPFC is the region of interest (ROI) in this study.

## Materials and Methods

### Participants

Twenty-two healthy volunteer participants (12 male; age range = 21–27 years, mean age = 22.7 years) were recruited from Hiroshima University. All participants were right-handed, with normal or corrected-to-normal vision. All were free of neurological and psychiatric disorders. To control possible confounding factors to brain activity (Duncan and Northoff, 2012), participants who were habitual drinkers or taking medication were excluded. Participants were not permitted to smoke tobacco from 3 h before the experiment started. Written informed consent was obtained from each participant before the investigation, in line with a protocol approved by the Research Ethics Committee of Hiroshima University. Each participant was paid a small fee for participating.

### Self-report measures

Early life stress was quantified using a CATS (Sanders and Becker-Lausen, 1995). The CATS is a 38-item questionnaire that measures subjective reports of various forms of childhood physical, sexual, and maltreatment abuse. For each item (e.g., “Did your parents ridicule you?”, “Did you ever seek outside help or guidance because of problems in your home?”, “Were you expected to follow a strict code of behavior in your home?”), participants rated how frequently a particular abusive experience occurred to them during their childhood and adolescence, using a scale of 0–4 (0 = never, 4 = always). The CATS score was calculated by summing the ratings after reversing the scores of reverse items. Sanders and Becker-Lausen (1995) reported strong internal consistency (Cronbach’s alpha = 0.90) and test–retest reliability (*r* = 0.89) for the total score. Validity was confirmed by demonstrating significant correlation with consequent psychological outcomes such as dissociation, depression, anxiety, difficulties in interpersonal relationships, and victimization, all of which have previously been associated with ELS (Sanders and Becker-Lausen, 1995; Kent and Waller, 1998). Numerous earlier studies have used this scale to assess ELS (e.g., Cohen et al., 2006; Philip et al., 2013b).

In addition to the CATS, we used the LES (Sarason et al., 1978) to assess recent stress levels. For the LES, participants were asked to indicate which of 57 events (e.g., “Death of close friend,” “Trouble with in-laws,” “Being fired from job”) occurred during the prior 12 months and to rate the impact of each event using a seven point scale, ranging from extremely negative (−3) to extremely positive (+3). The LES scores were calculated using summing impact ratings for all events. Sarason et al. (1978) reported significant test–retest reliabilities for the total score (*r* = 0.63 and *r* = 0.64) from the two test–retest reliability studies.

### Measurement of serum cortisol

To assess a possibility that the MPFC activation was altered by stress-elevated cortisol level, we measured cortisol levels in the blood. First, 3 ml venous blood was collected using anticoagulant-free vacuum tubes and kept at room temperature for 1 h with subsequent centrifugation at 2,000 × *g* for 20 min at 4°C. Serum was collected and stored at −80°C until use. Cortisol levels were measured by radioimmunoassay at SRL Corp. (Tokyo, Japan).

### Resting states

After NIRS probe placement, participants were seated on a comfortable chair facing a computer screen in a dark shielded room. Before the experimental tasks, participants performed counterbalanced resting EC and EO baseline periods of 5 min each. Each participant was instructed to relax and allow the mind to disengage during these periods. During the EO resting state, participants were asked to gaze at a fixation cross presented at the center of the computer screen.

### Cognitive task

After resting-state recording, participants performed cognitive tasks of two types: color-similarity judgment and color-preference judgment. Twenty-four colors were used in both tasks. Three colored squares were presented in each trial (see Figure 1A for example). The colored square presented at the upper center was the target color. The squares presented at the lower left and right were choices. The color squares were all 90 × 90 pixels. The similarity of colors was defined by the distance in CIELAB color space in which values L^∗^ (light–dark), a^∗^ (red–green), and b^∗^ (yellow–blue) are shown at right angles to each other to form a three-dimensional coordinate system. One color wheel of the a^∗^–b^∗^ plane was used to select color and to make color sets. Twelve colors were selected from one color wheel at every 30° of difference (see Figure 1B).

In both the tasks, color sets of two types (similarity-easy and similarity-difficult sets) were used (see Figure 1B). In the similarity-easy set (60 vs. 150°), one target-choice pair was clearly more similar (i.e., the difference between the target and choice was 60°) than another target-choice pair (i.e., the difference between target and choice is 150°). In the similarity-difficult set (90 vs. 120°), the similarities between the target and choices were closer between the two target–choice pairs. All target–choice color pairs were presented once in each of the tasks. The same color sets for 24 trials were used in these two task conditions.

In the color-similarity judgment task, participants were asked to judge which choice was more similar to the target color by pressing the button on the corresponding side. Participants were instructed clearly that the lightness was equal among these three colors. In the color-preference judgment task, participants were asked to judge which color pair (target–choice pair) they prefer. Participants were clearly instructed that no objectively correct answer exists: they must make their own decisions. These tasks were used in previous studies (Johnson et al., 2005; Nakao et al., 2013).

Participants performed eight blocks of six trials of tasks (four blocks per task). The block order was randomized across participants. Each block included a 10 s pre task baseline, 30 s cognitive task, and 20 s post task baseline (see Figure 1A). During the cognitive task, each trial began with the presentation of a task indicating a cue (“Similarity” or “Preference”) and three black squares indicating the three color square locations. One second later, color stimuli were presented for 4 s. Participants were instructed to press either the left or right button with the corresponding index fingers as quickly and accurately as possible after the stimuli were presented. The reaction time (RT) from the presentation of the color stimuli to the response was recorded. The presentation side of colors and the order of the trials were randomized across participants.

### NIRS data acquisitions

Relative changes in the concentration of oxy-Hb and deoxy-Hb were measured using a multichannel NIRS imaging system (FOIRE-3000; Shimadzu Corp., Japan) using three wavelengths (780, 805, and 830 nm) of infrared light based on Matcher et al. (1995). The data sampling time was 115 ms. The source–detector probes were placed in fronto-temporal regions. The probe set was mounted on a cap for fixation (Figure 1D). The lower frontal probes were positioned along the Fp1–Fp2 line according to the international 10–20 system used for electroencephalography. The distance between pairs of source–detector probes was set at 3 cm. Each measuring area between the pairs of source–detector probes was defined as a channel. It is considered that the machine with source–detector spacing of 3 cm measures points at 2–3 cm depth from the scalp (i.e., measurements are taken from the surface of the cerebral cortex; Hock et al., 1997; Toronov et al., 2001; Okada and Delpy, 2003a,b). The exact optical path length was unknown. Therefore, the unit used to measure these values was molar concentration multiplied by length (mM × mm). The 43 measuring points were labeled as ch1–ch43 (see Figure 1C). Because of a technical problem, data of three channels (ch25, ch28, and ch41) from eight participants failed to record a signal. Three-dimensional locations of the NIRS probe were measured using a Fastrak System (TX-2; Polhemus, USA). Using the MATLAB toolbox NFRI functions^1^, statistical results for each channel were shown on the surface of a standardized brain (Singh et al., 2005).

### NIRS analysis

The NIRS data analysis was done using software (MATLAB 8.0; The MathWorks Inc., Natick, MA, USA).

#### Resting-state data

Resting-state oxy-Hb data were filtered using a low-pass filter of 0.4 Hz. The linear trend caused by drift was removed (Tachtsidis et al., 2004). A Fast Fourier Transform (FFT) was performed on oxy-Hb data EC and EO resting-state data. The Welch technique with a Hanning window of 1024 sample points (117.76 s sliding window) and an overlap of 512 points was used. Power spectral density (mM × mm^2^/Hz) was calculated for each channel over the range of 0.02–0.15 Hz. Subsequently, the band-limited power in the following two frequency bands was calculated based on previous studies (Obrig et al., 2000; Tachtsidis et al., 2004; Näsi et al., 2011; Pierro et al., 2012): VLFO (0.02–0.04 Hz) and LFOs (0.04–0.15 Hz).

#### Cognitive task data

Oxy-Hb data during cognitive tasks were filtered using a low-pass filter of 0.2 Hz. The global drift was removed by application of a wavelet minimum description length (MDL) detrending algorithm (Jang et al., 2009) implemented in the MATLAB toolbox NIRS-SPM^2^(Ye et al., 2009). We specifically examined Δoxy-Hb, which is the most sensitive parameter of cerebral blood flow (Strangman et al., 2002). Many previous NIRS studies calculated a *z* score in each recording channel for comparison among participants (Minagawa-Kawai et al., 2002; Takeda et al., 2007; Shimoda et al., 2008; Matsuzawa, 2012). For this study, the *z* score at each channel was calculated as follows: the mean Δoxy-Hb value during the 30 s cognitive task vs. that during a 10 s pre task baseline period was divided by the standard deviation (SD) of Δoxy-Hb during the pre task baseline. The *z* scores in each task condition were averaged across blocks.

### Correlation analysis

Pearson correlation coefficients were calculated among possible combinations of our data (i.e., CATS score, LES score, cortisol levels, VLFO and LFO power spectrum density during EC and EO resting state of each NIRS channel, *z* scores for color-similarity judgment, and color-preference judgment of each NIRS channel, behavioral data during the two cognitive tasks). Outliers of each datum were excluded from the correlation analysis using an upper limit of the mean ± 3 SD of the participants’ data. *P* < 0.05 was considered a significant correlation. A bootstrap procedure (Efron and Tibshirani, 1986) with *n* = 1000 resamples was used to establish the 95% confidence intervals (CI) around the *r* value. In cases where we examine correlations with the CATS score, partial correlations were also calculated to exclude the possible effects from the LES score and cortisol level. When we test for significant differences between two correlation coefficients, Fisher’s *z*-transformation was applied to the correlation coefficients to generate a normal distribution. Then, *t*-statistics were calculated (Cohen and Cohen, 1983).

## Results

### Self-report and cortisol data

Table 1 presents a summary of the averaged self-report and cortisol data, and correlation coefficients among these measurements. The mean CATS score was 30.77 (SD = 12.91, range = 16–60). The mean LES score was 0.62 (SD = 2.65, range = −12 to 4). The mean blood cortisol level was 11.30 μg/dl (SD = 3.48, range = 4.7–17.8). None of these measurements was significantly correlated with age, gender, smoking status, history, or body mass index (BMI). The CATS score was not correlated with the LES score (*r* = 0.30, *p* = 0.18, CI = −0.07 to 0.59) as the index of recent stress. A clear distinction between early and recent life stress, measurement of ELS in CATS, is not confounded by recent life stress (LES). Both CATS (*r* = −0.04, *p* = 0.87, CI = −0.43 to 0.38) and LES (*r* = 0.15, *p* = 0.51, CI = −0.30 to 0.45) scores were not correlated with cortisol levels, suggesting that both early and recent life stress was not associated with the cortisol level, the elevation of which can alter the MPFC activity.

**Table 1:** **Summary of averaged self-report and cortisol data, and correlation coefficients (*r*) among these measurements**.

		CATS	LES	Cortisol (μg/dl)
*M* (SD)		30.77 (12.91)	0.62 (2.65)	11.30 (3.48)
*r*	CATS	1.00	0.30	−0.04
	LES	0.30	1.00	0.15
	Cortisol	−0.04	0.15	1.00

*M, mean; SD, standard deviation; CATS, child abuse and trauma scale; LES, life event stress scale*.

### Resting-state data

#### Resting-state power spectrum density

Table 2 shows averaged power across all NIRS channels for each resting-state condition (EC and EO) and for each frequency band (VLFO and LFO). The mean VLFO power of the EC resting state was 0.05 mM × mm^2^/Hz (SD = 0.02); that of the EO resting state was 0.07 mM × mm^2^/Hz (SD = 0.06). The mean LFO power of the EC resting state was 0.008 mM × mm^2^/Hz (SD = 0.004). That of the EO resting state was 0.01 mM × mm^2^/Hz (SD = 0.006). In both frequency bands, the EO resting state showed significantly greater power than the EC resting state showed [VLFO, *t*(21) = 2.15, *p* = 0.04; LFO, *t*(21) = 2.98, *p* = 0.007]. These results resemble those reported from earlier studies (Obrig et al., 2000; Tachtsidis et al., 2004; Yan et al., 2009).

**Table 2:** **Summary of averaged power (mM × mm^2^/Hz) across all NIRS channels for each resting-state condition (EC and EO) and for each frequency band (VLFO and LFO)**.

		EC	EO
VLFO	*M* (SD)	0.050 (0.020)	0.070 (0.060)
LFO	*M* (SD)	0.008 (0.004)	0.010 (0.006)

*M, mean; SD, standard deviation; EC, eyes-closed resting state; EO, eyes-open resting state; VLFO, very low-frequency oscillations; LFO, low-frequency oscillations*.

#### Correlation between resting state (VLFO, LFO) and early life stress (CATS score)

**Figure 2:**
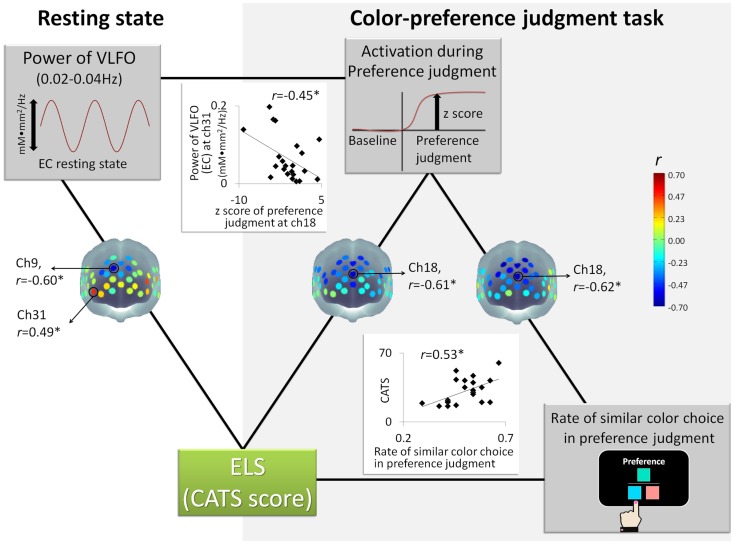
Schematic figure of each measurement and correlation results: *represents statistically significant *r* value (*p* < 0.05); ELS stands for early life stress; CATS denotes the Child Abuse and Trauma Scale; EC denotes eyes closed; VLFO denotes very low-frequency oscillations.

The power of VLFO during the EC resting state at the MPFC around ch9 (BA9) was negatively correlated with the CATS score (*r* = −0.59, *p* = 0.004, CI = −0.81 to −0.25; see Figure 2). In contrast, the power of VLFO at the lateral part of the lateral prefrontal cortex (LPFC) at ch31 (BA10) (*r* = 0.49, *p* = 0.02, CI = −0.03 to 0.75) and ch20 (BA 46) (*r* = 0.45, *p* = 0.04, CI = 0.002–0.78) was positively correlated with the CATS score (see Figure 2). When the effects from LES and cortisol level were excluded by partial correlation analysis, ch9 (*r* = −0.54, *p* = 0.02, CI = −0.87 to −0.16) and ch31 (*r* = 0.51, *p* = 0.03, CI = −0.17 to 0.81) showed similar results with significant correlation. Regarding the EO resting state, although the power of VLFO at the LPFC showed positive correlation with the CATS score (ch31, *r* = 0.49, *p* = 0.03, CI = 0.10–0.78), partial correlation *r* = 0.55, *p* = 0.02, CI = −0.19 to 0.85), the MPFC showed no correlation with the CATS score.

In contrast to the VLFO, the power of resting state LFO showed no significant correlation with the CATS score during either EC or EO. As additional statistical tests for the correlations between the CATS and EC resting state (VLFO, LFO), we compared the correlation coefficient of VLFO directly with that of LFO. A significant difference was found between these correlations [ch9, *t*(19) = 27.26, *p* < 0.001; ch31, *t*(19) = 2.88, *p* = 0.009]. These results suggest that ELS is specifically related to VLFO rather than LFO.

### Cognitive task data

#### Behavioral data

Table 3 presents behavioral data obtained for each task and each stimulus-set condition. The mean RT for the color-similarity judgment task was 1270.06 ms (SD = 310.32). That for the color-preference judgment task was 1612.80 ms (SD = 371.76). Within the color-similarity judgment task, the mean RT for the similarity-easy set trial was 1210.42 ms (SD = 324.19). That for the similarity-difficult set trial was 1329.70 ms (SD = 320.66). Within the color-preference judgment task, the mean RT for the similarity-easy set trial was 1581.74 ms (SD = 345.82). That for the similarity-difficult set trials was 1643.86 ms (SD = 429.97). Two-way repeated-measures ANOVA (task × stimulus set) revealed significant main effects of task [*F*(1, 21) = 37.80, *p* < 0.0001] and the stimulus set [*F*(1, 21) = 7.27, *p* = 0.01]. This result was consistent with those of previous studies using the same tasks (Johnson et al., 2005; Nakao et al., 2013).

**Table 3:** **Summary of behavioral data and averaged *z* score cross all NIRS channels for each task and each stimulus-set condition**.

		Similarity judgment task			Preference judgment task		
	
	
		All trials	Similarity– easy set trials	Similarity– difficult set trials	All trials	Similarity– easy set trials	Similarity– difficult set trials
RT	*M* (SD)	1270.06 (310.32)	1210.42 (324.19)	1329.70 (320.66)	1612.80 (371.76)	1581.74 (345.82)	1643.86 (429.97)
Error rate	*M* (SD)	0.27 (0.06)	0.14 (0.11)	0.40 (0.07)	–	–	–
Rate of similar color choice	*M* (SD)	–	–	–	0.50 (0.09)	0.49 (0.17)	0.50 (0.10)
	*r with CATS*	–	–	–	0.53	0.59	0.01
*z* Score (all channels)	*M* (SD)	0.25 (3.96)	0.13 (3.54)	0.46 (4.42)	0.01 (2.77)	−0.35 (3.21)	0.08 (2.76)

*M, mean; SD, standard deviation; r, Pearson’s correlation coefficient; CATS, child abuse and trauma scale; RT, reaction times*.

The mean error rate in the color-similarity judgment task was 0.27 (SD = 0.06). Within the similarity judgment task, the similarity-easy set trials (mean error rate = 0.14, SD = 0.11) showed significantly lower error rates than the similarity-difficult set trials (mean error rate = 0.40, SD = 0.07) [*t*(21) = 8.81, *p* < 0.001]. The observation of lower error rates further confirms that the similarity-easy set trials were indeed easier for our participants.

In addition to the difficulty in color-similarity judgment, color similarity might be yet another confounding influence, especially for color-preference judgment. The judgment of internal or subjective preference might be confounded by the more external or objective color similarity. It is possible that color-preference judgment can be biased by the color-similarity as the external criteria, especially when the color-similarity is a salient external figure (i.e., in the similarity easy–easy set trials). We therefore calculated the rate of similar color choice in the color-preference judgment task to assess how often the color-similarity biases color-preference judgment. We counted the trials in which a participant chose similar color in the color-preference judgment task; then that number was divided by the total number of color-preference judgment trials: 24. The mean rate of similar color choice in the preference judgment task was 0.50 (SD = 0.09). No significant difference was found between the similarity-easy set trial (mean rate of similar color choice = 0.49, SD = 0.17) and the similarity-difficult set trial (mean rate of similar color choice = 0.50 SD = 0.10). These mean rates of similar color choice are equal or almost equal to the chance level: no evidence shows that the judgment of internal or subjective preference was confounded by the external color similarity as a whole participant group, even when the color-similarity is a salient external figure (i.e., even in similarity-easy set trials).

The rate of similar color choice in the color-preference judgment task showed, however, a significant correlation with the CATS score (*r* = 0.53, *p* = 0.01, CI = 0.12–0.80, see Figure 2; Table 3). Consistent results were observed even when the effects of the LES score and cortisol level were excluded by partial correlation analysis (*r* = 0.49, *p* = 0.03, CI = −0.83 to 0.84). Within the color-preference judgment task, the rate of similar color choice in similarity-easy set trials showed a significant correlation with the CATS score (*r* = 0.59, *p* = 0.004, CI = 0.16–0.79; partial correlation, *r* = 0.65, *p* = 0.003, CI = 0.32–0.86). In contrast, no significant correlation was found in the similarity-difficult set trial (*r* = 0.01, *p* = 0.95, CI = −0.48 to 0.43; partial correlation, *r* = −0.20, *p* = 0.42, CI = −0.78 to 0.36). For further statistical tests for the correlations, we compared the correlation coefficient of the similarity-easy set trial directly with that of the similarity-difficult set trial. A significant difference was found between these correlations [*t*(19) = 2.51, *p* = 0.02]. The statistically significant difference underscores that the participants with high ELS tended to choose similar color in the color-preference judgments only when color-similarity was a salient external feature, which suggests a shift from internally to externally guided decision making in the similarity-easy set trials by experiencing ELS.

Other behavioral data showed no significant correlation with the CATS score.

#### NIRS data

Table 3 shows the averaged *z* score across all NIRS channels for each task and each stimulus-set condition. Regarding the *z* score of oxy-Hb for the cognitive tasks, the averaged *z* score across all channels for the color-similarity judgment task was 0.25 (SD = 3.96). That for the color-preference judgment task was 0.01 (SD = 2.77). Within the color-similarity judgment task, the *z* score for the similarity-easy set trials was 0.13 (SD = 3.54). That for the similarity-difficult set trials was 0.46 (SD = 4.42). Within the color-preference judgment task, the *z* score for the similarity-easy set trials was −0.35 (SD = 3.21). That for the similarity-difficult set trials was 0.08 (SD = 2.76). Because of the high SDs of the *z* score, three-way repeated-measures ANOVA (task × stimulus sets × channels) revealed no significant differences.

Nevertheless, the *z* score for the color-preference judgment task around ch18 (BA10) was negatively correlated with the CATS scores (ch 18, *r* = −0.61, *p* = 0.002, CI = −0.86 to −0.28, see Figure 2). Even when the effects from LES and cortisol level were excluded by the conduct of partial correlation analysis, consistent correlation results were found (ch18, *r* = −0.56, *p* = 0.01, CI = −0.82 to 0.08). In contrast, for the color-similarity judgment task, no significant correlation was found between the *z* score and the CATS score (ch 18, *r* = −0.14, *p* = 0.55, CI = −0.54 to 0.38; partial correlation, *r* = −0.22, *p* = 0.38, CI = −0.70 to 0.38). As further statistical tests for the correlations between the CATS and *z* score of cognitive tasks, we compared the correlation coefficient of the color-preference judgment task directly with that of color-similarity judgment task. A significant difference was found between these correlations [ch18, *t*(19) = 3.12, *p* = 0.006]. This result suggests that the degree of MPFC activation during color-preference judgment task was specifically related to ELS, as distinguished from the color-similarity judgment task.

The *z* score for the color-preference judgment task around ch18 was negatively correlated with the rate of similar color choice in the preference judgment task (ch18, *r* = −0.62, *p* = 0.002, CI = −0.85 to −0.23, see Figure 2). No significant correlation was found between the *z* scores for the color-similarity judgment task and the rate of similar color choice in the color-preference judgment task (ch 18, *r* = −0.21, *p* = 0.36, CI = −0.60 to 0.21). For further statistical tests for the correlations between the rate of similar color choice in the color-preference judgment task and the *z* score of cognitive tasks, we compared the correlation coefficient of color-preference judgment task directly with that of the color-similarity judgment task. Significant difference was found between these correlations [ch18, *t*(19) = 2.76, *p* = 0.01], which suggests that participants who showed decreased MPFC activation during color-preference judgment tend to make color-preference judgments based on color-similarity as an external criterion.

Within the color-preference judgment task, the rate of similar color choice in similarity-easy set trials showed significant correlation with the *z* score for the color-preference judgment task around ch18 (*r* = −0.57, *p* = 0.006, CI = −0.83 to −0.09). No significant correlation was found in the similarity-difficult set trial (ch 18, *r* = −0.20, *p* = 0.36, CI = −0.55 to 0.27). For further statistical tests for the correlations between the *z* score and the rate of similar color choice in the color-preference judgment task, we compared the correlation coefficient of the similarity-easy set trials directly with that of the similarity-difficult set trial. A significant difference was found between these correlations [*t*(19) = 2.17, *p* = 0.03].

Regarding the relation between the resting-state power spectrum density and the *z* scores of the color-preference judgment task, we calculated the correlation between the channels, which showed significant correlation with the CATS score (i.e., ch9 and ch31 of the EC resting state, and ch18 of the color-preference judgment task). The power of VLFO during the EC resting state at ch31 was negatively correlated with the *z* score of the color-preference judgment at ch18 (*r* = −0.45, *p* = 0.03, CI = −0.72 to 0.21, see Figure 2). The power at ch9 showed no significant correlation with the *z* score at ch18. This relation was not found between VLFO during the EC resting state and the *z* scores of color-similarity judgment. The LPFC is known to be activated consistently during goal-directed tasks (Cabeza and Nyberg, 2000; Fox et al., 2005; Owen et al., 2005; Kim et al., 2010). Moreover, it is temporally anti-correlated with midline regions (e.g., MPFC), such that resting-state activation within the MPFC is associated with attenuation of the LPFC (Fox et al., 2005, 2009). Based on these notions, it is possible that participants who experienced ELS showed increased baseline/spontaneous activations in the LPFC, and activations associated with the decrease of MPFC activation during self-oriented task via anti-correlative relation between these regions.

Taken together, as Figure 2 shows, the CATS sores were correlated with the VLFO power of EC resting state, the *z* score of color-preference judgment, and the rate of similar color choice in the color-preference judgment task. The following two tripartite relations were found. One is among the CATS score, the *z* score of the color-preference judgment task at MPFC, and the power of VLFO during the EC resting state at the LPFC. Another is among the CATS score, the *z* score of the color-preference judgment task at MPFC, and the rate of similar color choice in the color-preference judgment task. Furthermore, regarding the rate of similar color choice in the color-preference judgment task, the tripartite relation was observed in the similarity-easy, but not in the similarity-difficult set trials.

## Discussion

The present study was undertaken to assess the relations between ELS and the MPFC function during a resting state and self-oriented task. As Figure 2 shows, the CATS score was negatively correlated with the activations of MPFC during the EC resting state and color-preference judgment task (i.e., *z* score as relative activation from baseline): participants who experienced a high degree of ELS showed decreased activation both in the resting state and self-oriented task. These relations were specific to VLFO during EC and *z* score of self-oriented task. These significant correlations remained even with LES score and cortisol level. In contrast, LFO during EC resting state, VLFO and LFO during the EO resting state, and the *z* score of the control task showed no correlation with the CATS score. These results demonstrate for the first time the specific relation between ELS and the MPFC activation during both the EC resting state (VLFO power) and self-oriented task. Additionally, we observed that both ELS and the MPFC activation during color-preference judgment were associated behaviorally with the rate of similar color choice in the preference judgment. These relations were observed only in the similarity-easy set trials, which suggests that participants who showed high ELS and decreased MPFC activation during the self-oriented task tend to make decisions based on a salient external criterion during the task, which requires decisions based on their own internal criteria (i.e., tend to choose an obviously similar color in the color-preference judgment task). Taken together, our neuronal and behavioral findings demonstrate that high ELS is related to lower MPFC activation during both rest and the self-oriented task. This is behaviorally manifested as an abnormal shift from internally to externally guided decision making, even in situations where internal guidance is required.

Previous reports of fMRI studies have described that ELS is associated with greater deactivation of DMN during a working memory task (Philip et al., 2013b), with decreased functional connectivity within the DMN during a resting state (Burghy et al., 2012; van der Werff et al., in press; Cisler et al., 2013; Wang et al., in press; Philip et al., 2013a). Although NIRS as our index of resting-state brain activity and type of cognitive task differed from fMRI-BOLD, as described in reports of previous studies, our results were consistent with those in that ELS is associated with the attenuated MPFC function during not only the resting state but also self-oriented task. By contrast, an opposite correlation was observed between ELS and the resting-state LPFC activity (Figure 2), supporting a notion from previous reports of some studies that MPFC is temporally anti-correlated with the lateral cortical region (Fox et al., 2005, 2009). Interestingly, it has been suggested that MPFC has a role in biasing decisions based on internal criteria (Volz et al., 2006; Nakao et al., 2010, 2012). Based on these notions, a possible explanation underlying these correlations is that participants with a high degree of ELS cannot shift to refer to their own internal criteria during self-oriented tasks because changes in their MPFC and/or their balance to the lateral regions do not allow them to make the shift from external to internal. They are stuck in the external, which makes it appear as though they avoid making decisions based on their own internal criteria to eliminate anxiety about one’s own decision. This possibility seems more plausible in light of the observations that ELS induces anxiety-related behaviors in adulthood (Kalinichev et al., 2002; Dalle Molle et al., 2012).

**Figure 3:**
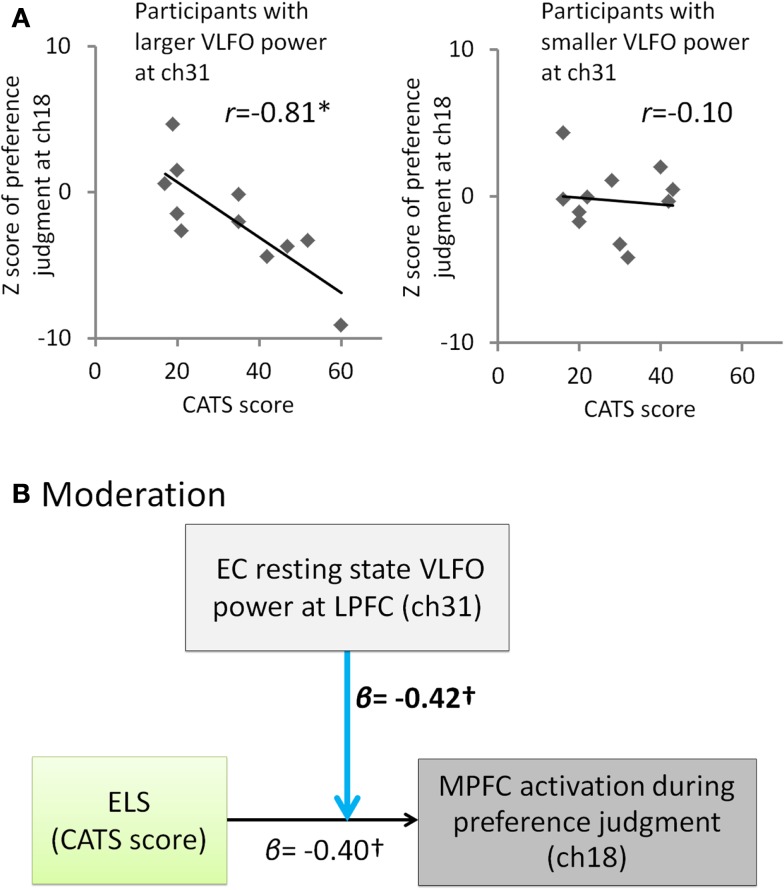
**Summary of the exploratory correlation and regression analyses for the relation among ELS, EC resting state VLFO at LPFC, and MPFC activity during color-preference judgment**. **(A)** Scatter plots and correlation coefficients between the ELS (CATS score) and the MPFC activation during the color-preference judgment (*z* score at ch18) for each of the subgroups divided by the median of the EC resting-state VLFO power at ch31. **(B)** Path model in which the effect from the ELS to the MPFC activation during the color-preference judgment was moderated by the LPFC resting-state activity. β represents standardized regression coefficient. *represents a statistically significant β value (*p* < 0.05). stands for marginally significant β value (*p* < 0.10). Light blue arrows indicate a marginally significant moderation effect. Dashed arrow represent not significant β value (*p* > 0.10). ELS is the early life stress. CATS stands for the Child Abuse and Trauma Scale. EC denotes eyes closed. VLFO denotes very low-frequency oscillations. MPFC denotes medial prefrontal cortex. LPFC denotes lateral prefrontal cortex.

Given that ELS disrupts a balance between LPFC and MPFC function leading to biasing decisions based on internal criteria during the self-oriented task, it is of interest to note that significant negative correlation between the ELS and the MPFC activation during self-oriented task was observed only in participants with enhanced LPFC activity during EC resting state (participants with larger VLFO power than median at ch31, *r* = −0.81, *p* = 0.003, CI = −0.94 to −0.54; participants with smaller VLFO power than median at ch31, *r* = −0.10, *p* = 0.77, CI = −0.66 to 0.55) (Figure 3A). This result suggests the possibility that whether the ELS affects to the MPFC activation during color-preference judgment was moderated by the resting state VLFO power at LPFC. To test this possibility, we conducted moderation analysis. This analysis revealed a marginal moderation effect from resting-state LPFC activity to the relation between the ELS and MPFC activity during color-preference judgment [moderation effect, β = −0.42, *t*(18) = −1.89, *p* = 0.08, see Figure 3B; effect of ELS, β = −0.40, *t*(18) = −2.01, *p* = 0.06; effect of resting-state LPFC activity, β = 0.005, *t*(18) = 0.02, *p* = 0.98; overall model statistics, *adjusted R*^2^ = 0.42, *F*(3, 18) = 6.08, *p* < 0.05]. These results suggest that higher ELS result in the decreased MPFC activation during the self-oriented task in the participants who showed enhanced resting-state activity at the LPFC.

**Figure 4:**
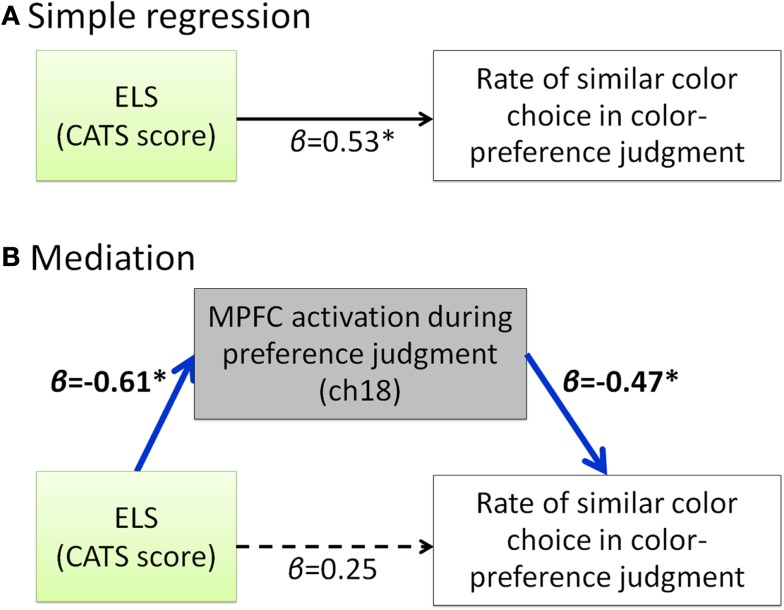
**Summary of the exploratory regression analyses about the relation among ELS, MPFC activity during color-preference judgment and the rate of similar color choice in color-preference judgment**. **(A)** Path model between the ELS and the rate of similar color choice in color-preference judgment. **(B)** Path model in which the effect from the ELS to the rate of similar color choice in color-preference judgment is mediated by the MPFC activity during the color-preference judgment. β represents the standardized regression coefficient. *represents statistically significant β value (*p* < 0.05). Dark blue arrows indicate the significant mediation effect. Dashed arrow represent not significant β value (*p* > 0.05). ELS denotes early life stress. CATS denotes the Child Abuse and Trauma Scale. MPFC denotes medial prefrontal cortex.

Our hypothesis that participants with a high degree of ELS cannot make a shift from external to internal during the self-oriented task is based on the result of positive correlation between the ELS and the rate of similar color choice in preference judgment (Figure 2). Considering that these two scores are both negatively correlated with the MPFC activity during the self-oriented task, it is possible to assume that the MPFC activity during color-preference judgment mediates the relation between ELS and the rate of similar color choice in color-preference judgment. For further exploratory analysis, we did mediation analysis to examine whether the relation between the ELS and the rate of similar color choice in color-preference judgment was mediated by the MPFC activity during color-preference judgment. The direct path (β = 0.53, *p* < 0.05, Figure 4A) from the ELS to the rate of similar color choice in color-preference judgment was significantly mediated by the MPFC activity during color-preference judgment (Sobel-test, *Z* = 1.73, *p* = 0.04, one tailed, Figure 4B). After controlling for the MPFC activity during color-preference judgment, the direct path between the ELS and the rate of similar color choice in color-preference judgment was no longer significant [β = −0.25, *t*(19) = −2.12, *p* = 0.28].

Taken together, it is feasible that the participants with a high degree of the ELS cannot shift to refer to their own internal criteria during self-oriented tasks because their MPFC does not allow them to make the shift from external to internal, especially in the case that the resting-state activity in LPFC was enhanced. Additional studies with more participants’ data are expected to confirm these preliminary findings from the multiple regression analyses.

How are our findings related to the self? Color-preference judgments presuppose an internal criterion according to which the judgment is made. The color must therefore be related and compared to an internal criterion (rather than an external criterion as in color-similarity judgment). Such relating and comparing must presuppose some kind of internal standard which is usually assumed to be the self. This process of relating and comparing to an internal criterion can thereby be described as self-related processing (see Northoff et al., 2006; Qin and Northoff, 2011). Our data hint that such comparing and relating against an internal standard, the self, is deficient in participant with high ELS, which raises two questions related to mediating neuronal mechanisms and related to the presupposed concept of the self. Our data contribute to the first question. In addition to the task-related activity during color-preference judgment, ELS were predicted by the degree of resting-state activity. This suggests some kind of encoding (or representation) of self-related information (about the self) in the resting-state activity itself; this is well in line with previous findings that observed neural overlap (or even prediction) between resting-state activity and self-related activity (see Schneider et al., 2008; Qin and Northoff, 2011; Whitfield-Gabrieli et al., 2011; Nakao et al., 2012; Huang et al., in press). The present study contributes here in that it suggests this self-related information in the resting state to be susceptible to, at least in part, ELS that seems to affect the self (or better its encoding or representation in the resting state) directly. That leads us directly to the second question: what concept of self do we presuppose here? The resting-state activity itself, by definition, shows no kind of cognitive activity related to specific stimuli or tasks. It also shows no sensory, motor, or affective neural activity. Consequently, the self that is encoded or represented in the resting state cannot be described as sensorimotor self (see for instance Legrand, 2007), affective self (Panksepp, 1998; Damasio, 2010), cognitive self (see Damasio, 2010), or social self (Schilbach et al., 2012). Instead, the self that is encoded in the resting state and susceptible to early stressful life events must be described conceptually independent of any specific sensory, motor, affective, cognitive, or social contents. Instead it is apparently more like some kind of structure or organization that serves as the internal standard or reference for subsequent comparing and relating of stimuli like colors that is color preference. Accordingly, our findings suggest a structure or organization-based concept of self which is well compatible with approaches in both neurophilosophy (Northoff, 2013) and the concept of the ego in neuropsychoanalysis (see Northoff, 2011).

Despite the importance of these data for revealing the relation between ELS and brain function, these findings leave several questions unresolved. First, although we found several correlations, as shown in Figure 2, causal relations among these measurements remain unresolved. These results tempt us to advance the following hypothesis: ELS results in increased LPFC during rest and decreased MPFC during rest and self-oriented task later in life. Because of these characteristics of neural activities, people with ELS make decisions based on a salient external criterion even when they must make a decision based on their own internal criteria. This hypothesis, however, remains speculative in the absence of data to corroborate these causal relations. Although we obtained consistent results (see Figures 3 and 4) with this hypothesis, those were from the preliminary regression analyses. Animal studies manipulating ELS and measuring brain activity under similar experimental settings must be done to reveal the effects of ELS.

Second, no significant difference was found between color-similarity judgment and color-preference judgment in terms of the *z* score. A previous study using fMRI (Johnson et al., 2005) showed increased MPFC activation during the color-preference judgment task compared to the color-similarity judgment task. One possible reason for this discrepancy is that the NIRS measures only that activity occurring within the surface of the MPFC. The regions measured by NIRS in this study and those others showing significant difference between these tasks in previous studies might not be exactly the same regions. Nevertheless, we found a significant difference between color-preference and color-similarity judgment as the difference of correlation with the CATS score. This result lends further support to the notion that ELS is associated with attenuated or decreased MPFC function. However, we must be careful about this discrepancy to interpret the correlation result: it is possible that the regions showing a significant difference between these two tasks were dissociated from the regions showing correlation with the CATS score. Additional studies using fMRI must be undertaken to examine this possibility.

## Conclusion

This preliminary study was conducted to investigate the relations between ELS and the MPFC function during a resting state and self-oriented task. Our obtained NIRS data have revealed that ELS is associated with decreased activation within the surface regions of MPFC during rest and during the self-oriented task. In addition, ELS and the decreased activation within the MPFC during the self-oriented task was associated with a tendency to make a decision based on a salient external criterion during the self-oriented task. This study is expected to be of great interest in the field of ELS itself in that it provides evidence about the relations among ELS, resting-state brain activity, task induced brain activity, and behavioral tendencies. Beyond elucidating the phenomena associated with ELS, this line of investigation is expected to contribute to improvement of our understanding of resting-state brain activity and self-oriented processes. Because the present study confronts the two limitations as described above, additional human fMRI experiments and animal studies are expected to increase the validity of the findings presented herein.

## Conflict of Interest Statement

The authors declare that the research was conducted in the absence of any commercial or financial relationships that could be construed as a potential conflict of interest.

## Footnotes

^1^http://www.jichi.ac.jp/brainlab/tools.html

^2^http://bisp.kaist.ac.kr/NIRS-SPM.html
